# Do AI Chatbots Incite Harmful Behaviours in Mental Health Patients?

**DOI:** 10.1192/bjo.2024.225

**Published:** 2024-08-01

**Authors:** Harikrishna Patel, Faiza Hussain

**Affiliations:** Southern Health NHS Foundation Trust, Southampton, United Kingdom

## Abstract

**Aims:**

The contribution of mental illness towards total Disability Adjusted Life Years is increasing according to the Global Burden of Disease study. As the need for mental health services increases, technological advances are being deployed to improve the delivery of care and lower costs.

The emergence of Artificial Intelligence (AI) technology in mental health and companionship is an evolving topic of discussion. There have been increasing debates about the use of AI in managing mental health problems. As the AI technology and its use grows, it is vital to consider potential harms and ramifications.

There are very limited discussions about the use of chatbots and relevant AI by humans to commit crime especially in those suffering from mental illness. AI can potentially serve as an effective tool to misguide a vulnerable person going through a mental health problem e.g. encourage someone to commit a serious offence. There is evidence that some of the most used AI chatbots tend to accentuate any negative feelings their users already had and potentially reinforce their vulnerable thoughts leading to concerning consequences.

The objective of this study is to review existing evidence for harmful effects of AI chatbots on people with serious mental illness (SMI).

**Methods:**

We conducted a review of existing evidence in five databases for relevant studies. The search sources were 4 bibliographical databases (PsycINFO, EMBASE, PubMed, and OVID), the search engine “Google Scholar” and relevant grey literature. Studies were eligible if they explored the role of AI and related technology in causing harm in those with SMI.

**Results:**

Initial searches constrained the scope of review to the harmful effects of AI use in mental health and psychiatry and not just the association with crime due to very limited existing data.

**Conclusion:**

Whilst current AI technology has shown potential in mental healthcare, it is important to acknowledge its limitations. At present, the evidence base for benefits of AI chatbot in mental healthcare is only just getting established and not enough is known or documented around the harmful effects of this technology. Nevertheless, we are seeing increasing cases of vulnerable mental health patients negatively influenced by AI technology. The use of AI chatbots raises various ethical concerns often magnified in people experiencing SMI. Further research will be valuable in understanding the ramifications of AI in psychiatry. This will also help guide the developers of this important and emerging technology to meet recognised ethical frameworks hence safeguarding vulnerable users.

